# The Big Pet Diabetes Survey: Perceived Frequency and Triggers for Euthanasia

**DOI:** 10.3390/vetsci4020027

**Published:** 2017-05-14

**Authors:** Stijn J.M. Niessen, Katarina Hazuchova, Sonya L. Powney, Javier Guitian, Antonius P.M. Niessen, Paul D. Pion, James A. Shaw, David B. Church

**Affiliations:** 1 Diabetic Remission Clinic, Department of Clinical Science and Services, Royal Veterinary College, Hawkshead Lane, North Mymms, Hertfordshire AL9 7TA, UK; khazuchova@rvc.ac.uk (K.H.); dchurch@rvc.ac.uk (D.B.C.); 2 Institute of Cellular Medicine, Medical School Newcastle, Framlington Place, Newcastle-upon-Tyne NE2 4HH, Tyne and Wear, UK; jim.shaw@ncl.ac.uk; 3 E-Media Unit, Royal Veterinary College, Hawkshead Lane, North Mymms, Hertfordshire AL9 7TA, UK; spowney@rvc.ac.uk; 4 Veterinary Epidemiology and Public Health Group, Royal Veterinary College, Hawkshead Lane, North Mymms, Hertfordshire AL9 7TA, UK; jguitian@rvc.ac.uk; 5 Twan Consultancy, Romerstraat 24, 5911 HV Venlo, The Netherlands; apmniessen@hotmail.com; 6 Veterinary Information Network, 777 West Covell Blvd, Davis, CA 9561, USA; paul@vin.com

**Keywords:** diabetes mellitus, survey, euthanasia, quality of life, insulin injections

## Abstract

Current pet diabetes mellitus (DM) treatment necessitates the active daily involvement of owners and can be costly. The current study aimed to investigate the owner population which opts for euthanasia instead of DM treatment. A survey was designed using multiple feedback steps and made available online to veterinarians world-wide. A total of 1192 veterinarians completed the survey and suggested a median one in 10 diabetic pets are euthanased at diagnosis; a further median one in 10 within one year because of lack of success or compliance. Perceived most important motivating factors included “presence concurrent disease” (45% respondents); “costs” (44%); “animal age” (37%); “problems obtaining adequate control” (35%); “pet welfare” (35%); and “impact owner’s lifestyle” (32%). Cats in Canadian (odds ratio (OR) 2.7), Australian (OR 2.3), rural (OR 1.6) and mixed (OR 1.7) practices were more likely to be euthanased because of DM diagnosis, while cats presented to referral/university were less likely to be euthanased (OR 0.6). Dogs were more likely to be euthanased because of DM in Canadian (OR 1.8), rural (OR 1.8) and mixed (OR 1.6) practices. The survey results suggest that benefit exists in improved DM education with emphasis on offering a choice of treatment styles ranging from intense and expensive to hands-off and cheap.

## 1. Introduction

Owners’ active participation in the treatment is essential for successful management of diabetes mellitus (DM) in companion animals. The disease and the treatment commitments are likely to have considerable impact on owners’ daily routines and quality of life (QoL) and might represent a significant temporal, financial and emotional burden. Several areas of everyday life that are negatively influenced by DM and its treatment have been identified in a recent survey concerning the QoL of diabetic cats and dogs and their owners [1,2]. Owners’ perception of substantial impairment in one or more of these areas could potentially lead to treatment cessation and similar concerns could even prompt the decision to decline treatment at the time of diagnosis. However, the above mentioned QoL survey reflects the views only of those who had already accepted and initiated the treatment and the assumption that owners who decline or opt to cease treatment share the same concerns might not be correct. Rationale of the latter group is not well documented.

Epidemiologic data regarding DM and survival of dogs and cats with DM are limited [3,4] and only a few studies concerning diabetic cats treated at university teaching hospitals (UTHs) provide some insights to factors associated with mortality [5,6,7]. However, none of the studies have assessed the reasons for owners to decline or cease treatment. Moreover, most information stems from UTHs and large scale studies concerning treatment of DM in private practice are lacking.

The aim of this study was to document the perceived frequency of euthanasia among cats and dogs with DM at the time of diagnosis and within the first month and first year of treatment, investigate the possible influence of demographic factors on the decision to decline or cease treatment of DM as well as identify the owner’s reasons for making this decision and compliance issues. To obtain this information, the Big Pet Diabetes Survey was designed and performed among veterinary clinicians. At the same time, this approach allowed assessment of current DM treatment habits and experiences of participating clinicians.

## 2. Materials and Methods

### 2.1. Design of Survey

Qualitative research was conducted as part of the design phase of the Big Pet Diabetes Survey. To ensure all items were DM-treatment-centred and of specific validity to clinicians, diabetic pets and their owners, detailed discussions were conducted with 65 veterinary surgeons and 36 nurses (both in primary practice and in referral practice, both in the UK and USA), as well as a clinical epidemiologist.

This initial phase resulted in the formulation of five demographic questions and 36 specific DM treatment questions, including quantification of the number of pets being denied treatment, reasons for such denial, treatment habits and success rates of the participating clinician, as well as perceived concerns of client and clinician surrounding DM treatment. All questions required the respondent to choose one multiple choice question answer or a numeric answer. A free comments section at the end of the survey provided the opportunity for additional comments.

The second phase consisted of the Big Pet Diabetes Survey being digitalised and publicised online using a software package (Questionmark Perception Manager, Questionmark, London, UK). An initial trial was conducted amongst 20 veterinary surgeons and 10 veterinary nurses in order to identify areas of confusion and assess the questions’ true validity. Feedback was used to fine-tune and finalise the questions and answer categories, before subsequent application of the final online version (Supplement 1) in the larger veterinary clinician community (third phase). The presented data were voluntarily provided by veterinarians in the full knowledge that this information facilitated the study of diabetic pets and their owners; the study was approved by the Royal Veterinary College’s ethics and welfare committee.

### 2.2. Recruitment of Respondents

The final digitalised Big Pet Diabetes Survey was made available online using the Uniform Resource Locator (URL) www.rvc.ac.uk/diabetesvet. Recruitment of respondents was conducted in 2008 and 2009, parallel with and through the same channels as the diabetic pet owner survey recruitment as described previously [1,2].

### 2.3. Statistical Analysis

Descriptive statistics were obtained for all variables using proportion of observations in different categories for categorical variables, mean and standard deviation (SD) for normally distributed quantitative variables and median and interquartile range (IQR) for non-normally distributed quantitative variables. Data regarding the diabetes treatment decision questions were analysed through univariate analysis using the non-parametric Kruskall–Wallis test for questions in the format “out of 10 cats/dogs/owners, how many...”. Multivariate analysis was performed to assess the same relationships while adjusting for potential confounding effects by selected demographical factors. For the purpose of reporting the results, the category “practice type I” includes mixed and 100% small animal practices, and “practice type II” includes referral/university, charity and private practices.

Continental Europe, suburban, 100% small animal and private practices, as well as practices with <20% of cats/animals insured served as reference category. Once the variables to be included in the model were identified, ordinal logistic regression was used.

In view of the low frequency of answers indicating four or more for questions in the format “out of 10 cats/dogs/owners, how many...” and the results of testing for parallel lines (testing for equal slope coefficients across outcome variables), answers options were re-grouped into five categories, specifically: 0, 1, 2, 3 and greater or equal to 4. Assessment of the multivariate analysis model was done by testing for parallel lines; the model was deemed appropriate if *p* > 0.05.

All statistical analyses were performed using a statistical software package (SPSS 17.0 for Windows, SPSS Inc., Chicago, IL, USA) and *p* ≤ 0.05 deemed significant.

## 3. Results

A total of 1192 veterinarians completed the survey. The majority of respondents worked in suburban and urban areas of the USA and UK and Ireland. Most of the clinicians worked in 100% small animal private practice settings, serving a predominantly uninsured patient population and diagnosed DM in less than six cats and less than six dogs each year (Table 1).

A summary of their DM treatment habits is provided in Table 2.

A total of 946 (93.2%) and 984 (95.3%) of the clinicians, respectively, reported they would “certainly” treat their own cat or dog with insulin injections should their pet be diagnosed with DM.

The proportions of cats and dogs euthanased at the time of DM diagnosis, those euthanased because of injection issues or those ceasing the treatment within 1 month and 1 year of DM diagnosis are given in Table 3.

The opinion of the majority of clinicians (percentage of clinicians in brackets) was that owners were “probably” (41.8%) or “definitely” (26.7%) more likely to opt for insulin injection therapy when animals were insured. Clinicians (percentage of clinicians in brackets) also assumed that owners were “probably” (31.2%) or “definitely” (57.5%) more likely to opt for insulin injections when they themselves or close family or friends were diabetic.

**Figure 1:**
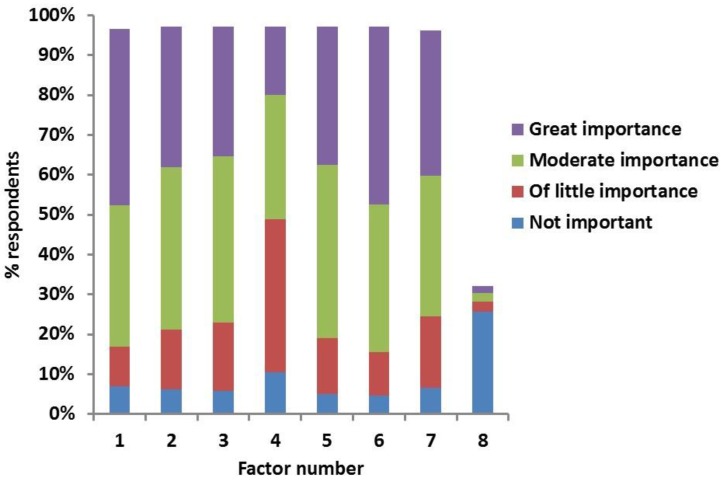
Clinicians’ answers to the survey question “If diabetic animals are euthanased/treatment is stopped/not started, how important are the following factors?” Factors indicated by numbers on X-axis; 1. costs; 2. welfare of pet; 3. too much impact on lifestyle of owner; 4. injection problems; 5. problems obtaining adequate control; 6. concurrent disease; 7. Age of the animal; 8. other (additional issues reported by the respondents and rated according to importance).

Factors considered by clinicians (percentage of clinicians in brackets) to be of “great importance” in the owner’s decision making to euthanase diabetic animals, stop the treatment or not start DM treatment in the first place were (in decreasing order) “concurrent disease” (45%); “costs” (44%); “age of the animal” (37%); “problems obtaining adequate control” (35%); “welfare of the pet” (35%); “too much impact on owner’s lifestyle” (32%); and “injection problems” (17%) (Figure 1).

**Figure 2:**
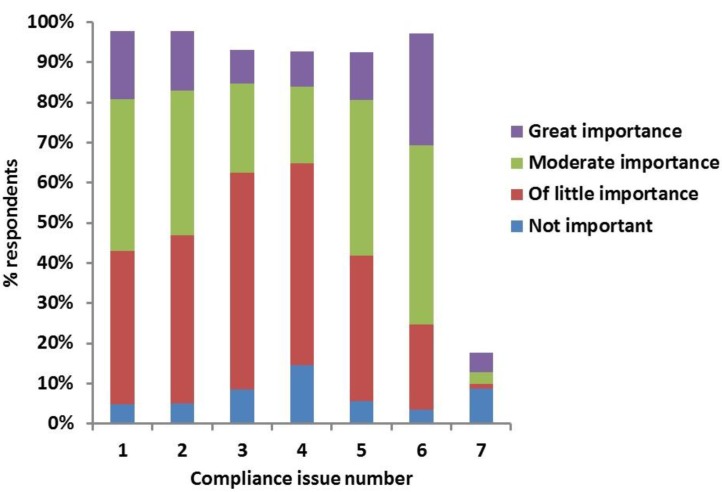
Clinicians’ answers to the survey question "According to you how important are the following compliance issues encountered in your practice?” Compliance issues indicated by numbers on X-axis: 1. **owner** having injection difficulties; 2. **cat** causing injection difficulties; 3. **dog** causing injection difficulties; 4. inappropriate insulin storage; 5. injections not given at right times; 6. owner does not stick to feeding protocol; 7. other (additional issues reported by the respondents and rated according to importance).

The most commonly encountered compliance issues considered to be “of great importance” by the clinicians (percentage of clinicians in brackets) were “owner does not stick to feeding protocol” (28%); “owner having injection difficulties” (17%); “cat causing injection difficulties” (15%); “injections not given at right times” (12%); “inappropriate insulin storage” (9%); and “dog causing injection difficulties” (8%) (Figure 2).

**Figure 3:**
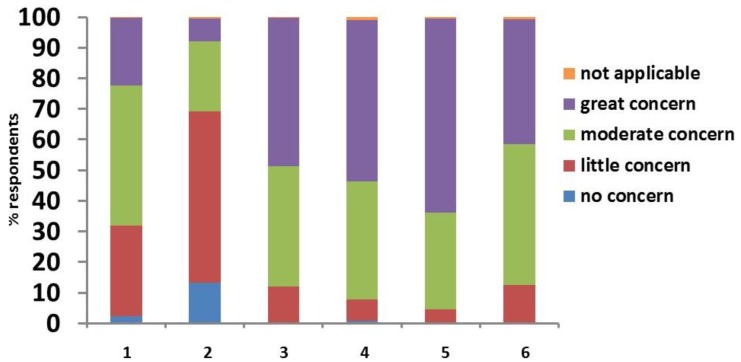
Clinicians’ answers to the survey question “According to you, how much are owners of diabetic animals concerned by the following issues?”: 1. hypoglycaemia; 2. diabetic ketoacidosis; 3. having to inject their animal; 4. costs of treatment; 5. quality of life of the animal; 6. lifestyle changes that the owner has to make.

Based on their experience, clinicians (percentage of clinicians in brackets) considered “QoL of the animal” (60%); “costs of treatment” (52%); “having to inject their animal” (48%); “lifestyle changes the owner has to make” (38%); “hypoglycaemia” (23%); and “diabetic ketoacidosis (DKA)” (7%) to be of “great concern” for owners of diabetic cats and dogs (Figure 3).

**Figure 4:**
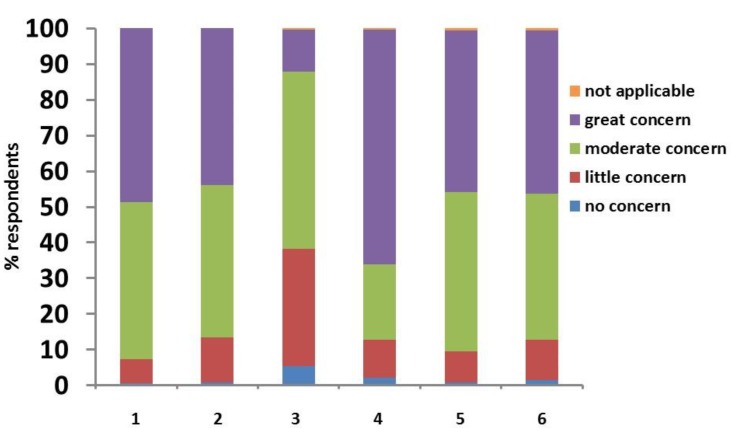
Clinicians’ answers to the survey question “How much do the following issues worry you as a vet?”: 1. hypoglycaemia; 2. diabetic ketoacidosis; 3. costs; 4. quality of life of the animal; 5. difficulties in obtaining rapid and adequate control; 6. difficulties of getting the owner on board with the treatment.

For the clinicians themselves, “QoL of the animal” (63%) was also the most commonly stated factor of “great concern”, followed by “hypoglycaemia” (46%), “difficulties in getting the owner on board with the treatment” (44%), “DKA” (43%), “difficulties in obtaining rapid and adequate control” (42%) and “costs of treatment” (13%) (Figure 4).

Information about clinicians’ views in regard to the QoL achieved by owners and their pets as well as information about frequency of complications such as hypoglycaemia and DKA is provided in Table 4.

### 3.1. Results of Univariate Analysis

The variables “country”, “location of the practice” and “practice type I” were found to be significantly associated with the answers to questions D/C 1 (“frequency of euthanasia of newly diagnosed diabetic dogs/cats at time of diagnosis”) and D/C 2 (“frequency of euthanasia of newly diagnosed diabetic dogs/cats at time of diagnosis because of not wanting to inject”), and “practice type II” was associated with the answers to question C 2. “Practice type I” was associated with the answers to questions D/C 3 and 4. Additionally, “practice location” and “practice type II” were associated with the answers to question D 3, while “% insured” was associated with the answers to question D 4. The results of univariate analysis are provided in Supplement 2.

### 3.2. Results of Multivariate Analysis

Results of multivariate analysis can be seen in Table 5 and Table 6.

“Percentage insured” (with a 20% cut-off) was found not to be significantly associated with answers to any of the four assessed questions (D/C 1, 2, 3, 4). Cats presented to practices in Canada (odds ratio (OR) 2.7) and Australia (OR 2.3) as well as in rural (OR 1.6) and mixed (OR 1.7) practices were more likely to be euthanased because of DM diagnosis, while cats presented to referral centres or UTH were at lower risk (OR 0.6). Dogs were more likely to be euthanased because of DM in Canada (OR 1.8) and if they were presented to rural (OR 1.8) and mixed (OR 1.6) practices. Cats and dogs presented to practices in Canada (OR for cats 2.4, OR for dogs 2.1) and to rural practices (OR for both cats and dogs 2.2) were at higher risk to be euthanized on owner request because of not wanting to treat with insulin injections; the risk was lower for cats presented to referral centres or universities (OR 0.54). Treatment of DM was less likely to be ceased within the first month of diagnosis because of lack of success or compliance in cats presented to practices in Canada (OR 0.5), USA (OR 0.6) and UK and Ireland (OR 0.5) and in dogs in UK and Ireland (OR 0.5). On the other hand, both cats and dogs were more likely to have DM treatment stopped within 1 month (OR for cats 2.2, OR for dogs 2.9) and within 1 year (OR for cats 2.0, OR for dogs 1.9) of DM diagnosis if presented to charity practices. Being presented to a mixed practice increased the risk for treatment cessation within 1 year in both cats (OR 1.6) and dogs (OR 1.7). The risk of treatment cessation within 1 year of DM diagnosis was also higher for cats presented to referral or university hospitals (OR 1.6).

## 4. Discussion

The Big Pet Diabetes Survey is the first of its kind to document perceptions of veterinarians on frequency and reasons for euthanasia of diabetic cats and dogs at the time of diagnosis and within the first year of treatment. Furthermore, the study also obtained information about DM treatment habits as well as clinician’s and perceived owner’s concerns.

The study revealed that according to the interviewed population of veterinarians, a median of one in 10 cats and dogs with DM were euthanased on request of the owner at the time of diagnosis and in another median of one in 10 cats and dogs, treatment was stopped within one year because of lack of response or compliance. Although the frequency of euthanasia within the first month of DM diagnosis in this study (0–1 of 10 cats and dogs) was comparable to previous reports in cats (mortality rates of 11%–17% within first 3–4 weeks) [5,7], higher figures were reported in a large Swedish insurance study concerning diabetic dogs [3]. In the latter investigation, 30% of dogs were euthanased at time of diagnosis and another 15% within 30 days. Mortality rates within 1 year of DM diagnosis were found to be 35%–40% in cats [4,5,7] and 60% in dogs in the above mentioned Swedish study [3], which is higher than in the present investigation. This might be attributable to demographic and temporal differences, previous studies concerning cats presented to UTHs in the USA and Switzerland [5,6,7] and primary-care veterinary practices in the UK [4] and diabetic dogs in Sweden [3].

In hindsight, there might have been advantage to re-phrasing the euthanasia-questions to state “out of 20” instead of “out of 10”, allowing for more detailed assessment of the most common answers, which were mostly around the one out of 10. Comparison of the current data with other studies or extrapolation to other diabetic pet populations should therefore be performed with caution, especially in view of the demonstrated interacting demographic characteristics. Higher proportions of euthanasia cases or treatment cessations were reported to occur amongst newly diagnosed cats and dogs in Canada and Australia, in rural, mixed and charity practices, and a lower proportion in referral or UTH. These findings could relate to differing client perceptions and expectations or attitudes amongst different countries and practice types, as well as possibly differing economic factors. Additionally, results of our study could also imply that should a greater proportion of respondents have originated from outside the USA, UK and Ireland or Continental Europe, and/or from rural, charity and mixed practices, a different picture might well have been recorded, with a trend towards more euthanasia and cases of premature cessation of treatment. Educational programmes focussed on offering a variety of DM management styles (ranging from intense and expensive to relatively hands-off) might positively affect these increased euthanasia rates, although the current dataset does not provide direct proof for this. Interestingly, despite the majority of clinicians suspecting clients to be more likely to opt for insulin treatment if their animal is insured, the insurance level did not seem to significantly influence treatment decisions.

When considering euthanasia, “concurrent disease”, “costs” and “age” were reported to be the most important factors for owners, whereas “injection problems” were considered to be the least important. The latter is also in alignment with the results of the concurrently performed QoL survey conducted among owners [1,2]. Older age was negatively associated with survival in a previous report [5] and cats with concurrent disease had 70% higher mortality rate in comparison to those without [7], substantiating the results of the current survey. The requirement for multiple practice visits, blood sampling and daily treatment administration associated with current treatment regimens would also justify the perceived importance of “costs”. Interestingly, “too much impact on owner’s lifestyle” was also considered of importance by most clinicians, which is in line with research among diabetic pet owners, where it featured dominantly among the top 10 items with negative impact on QoL constructed by owners themselves [1,2].

In general, there are only limited data available on compliance issues in veterinary medicine with most information extrapolated from human research [8]. Although it is generally accepted that a high level of owner and pet compliance is essential for successfully treating any disease and most particularly DM [9,10], no work has previously assessed the relative importance of specific compliance issues in managing canine and feline diabetics. The current data suggest most common compliance issues include adherence to feeding protocols and injection difficulties.

Quality of life of the animal was considered the most important concern in regard to DM for both owners and clinicians in this study, and based on clinicians’ experience, a small proportion of owners perceived the QoL of their animal or their own lifestyle to be unsatisfactory after starting the insulin treatment. “Costs of treatment” and “having to inject their animal” featured prominently on the list of owners’ concerns according to the experience of surveyed clinicians, which was in contrast with the owner data derived from the QoL survey [1,2]. This might represent a difference between populations of owners in these survey studies, and possibly also clinicians’ misconception of owners’ perceptions. Indeed, clinicians would have responded from their experience with both owners who proceed with long-term insulin injections (possibly similar to the QoL survey respondents) and those that have discontinued treatment in the short-term.

Clinicians themselves were more frequently concerned about hypoglycaemia and DKA, which might reflect their perception of these complications as being serious and possibly life-threatening. Hypoglycaemia was reported to occur in two out of 10 cats and dogs in the present study and one in 10 cats and dogs suffered from DKA. The latter complication was reported to have occurred in 12% and 34% of cats with DM presented to UTHs [6,7]. This represents further indications of remaining room for improvement in current treatment methods.

There are a number of limitations to this study. Information surrounding the frequency and reasons for the owner to opt for euthanasia or treatment cessation was gained by asking veterinarians, rather than obtaining this information from owners directly or by analysing veterinary practice records. Unfortunately, approaching a large number of such owners would have been difficult, since in most cases they were unlikely to have reasons to continue to attend their veterinary practice. Therefore, for such information to become available, it seemed a logical alternative to record the experiences of veterinarians dealing with both compliant and non-compliant diabetic animals and their owners on a regular basis.

Additionally, the possibility exists for this study to be affected by so-called recall bias. It could, for instance, be envisioned that this has led to lowering of the reported frequency of euthanasia when predominantly asking those clinicians with a positive past experience in treating diabetes (or vice versa). Indeed, most clinicians reported they would treat their own pet if diagnosed with diabetes, indicating such possible positive past experience. However, obtaining this type of information from medical records of veterinary practices on three different continents would have been extremely difficult. Some of the information could have been obtained from large database systems such as the Veterinary Medical Database (VMDB) in North America or VetCompass in the UK; however, the former would have introduced bias towards university teaching hospitals (shown to be a possible confounding factor in the current study) and the latter had just been launched at the time of performing this survey. Experience in working with the VetCompass database also reveals that accurate information surrounding reasons for euthanasia is infrequently recorded; nevertheless, a future study based on VetCompass should be considered. Records of insurance companies could have represented another alternative, however, would have been limited to an insured population.

Finally, the current study used a questionnaire consisting of closed-ended questions. This might have biased clinicians’ responses, despite offering the option “other” to provide additional information. However, the use of open-ended questions is associated with higher risk of larger item non-response or invalid answers resulting in missing data [11]. When analysing these free text contributions, no major and consistent misunderstandings or omissions were identified, confirming the validity of the initial design of the Big Pet Diabetes Survey. Therefore, although the limitations of the current approach should be kept in mind, the obtained data adds unique large-scale insight to the knowledge surrounding this topic.

## 5. Conclusions

In conclusion, the Big Pet Diabetes Survey records the perceived treatment cessation and euthanasia rates in companion animals diagnosed with DM amongst a large number of clinicians in a large number of countries. The causes behind country- and practice-type-associated differences should be further investigated. In view of the recorded perceived factors driving euthanasia instead of (continued) treatment, designing novel and more successful DM treatment protocols and options would be desirable; ideal treatment characteristics would include being less expensive, rapidly effective whilst carrying a low hypoglycaemia-risk and with decreased impact on owner lifestyle.

## Figures and Tables

**Table 1:** Country of origin of 1192 responding veterinary clinicians and breakdown according to various demographical parameters.

Country or Region Practicing Clinician	Number of Respondents (%)	Location	Practice Type I	Practice Type II	% Cats and Dogs Insured	DM Diagnoses/Year
USA	807 (67.8%)	Rural 95 (11.8%) Urban 192 (23.8%) Suburban 509 (63.1%) Other 11 (1.4%)	SA 715 (89.3%) Mixed 62 (7.7%) Other 20 (2.5%)	Private 732 (91.2%) Charity 9 (1.1%) Referral/Uni 37 (4.6%) Other 25 (3.1%)	<20% insured 789 (98%) >20% insured 16 (2%)	Cats: <6: 330 (40.9%) 6–10: 282 (34.9%) >10: 195 (24.2%) Dogs: <6: 492 (61.3%) 6–10: 195 (24.3%) >10: 115 (14.3%)
UK and Ireland	168 (14.1%)	Rural 26 (15.5%) Urban 72 (42.9%) Suburban 67 (39.9%) Other 3 (1.8%)	SA 139 (83.2%) Mixed 26 (15.6%) Other 2 (1.2%)	Private 129 (77.2%) Charity 20 (12%) Referral/Uni 15 (9%) Other 3 (1.8%)	<20% insured 59 (35.1%) >20% insured 109 (64.9%)	Cats: <6: 90 (53.6%) 6–10: 47 (28.0%) >10: 31 (18.5%) Dogs: <6: 97 (58.1%) 6–10: 43 (25.7%) >10: 27 (16.2%)
Canada	93 (7.8%)	Rural 10 (10.8%) Urban 27 (29.0%) Suburban 54 (58%) Other 2 (2.2%)	SA 80 (86%) Mixed 11 (11.8%) Other 2 (2.2%)	Private 82 (88.2%) Charity 3 (3.2%) Referral/Uni 4 (4.3%) Other 4 (4.3%)	<20% insured 90 (96.8%) >20% insured 3 (3.2%)	Cats: <6: 36 (38.7%) 6–10: 36 (38.7%) >10: 21 (22.6%) Dogs: <6: 67 (72.8%) 6–10: 19 (20.4%) >10: 6 (6.5%)
Continental Europe	93 (7.8%)	Rural 13 (14.1%) Urban 46 (50%) Suburban 32 (34.8%) Other 1 (1.1%)	SA 73 (78.5%) Mixed 16 (17.2%) Other 4 (4.3%)	Private 68 (73.1%) Charity 2 (2.2%) Referral/Uni 21 (22.6%) Other 2 (2.2%)	<20% insured 85 (92.4%) >20% insured 7 (7.6%)	Cats: <6: 48 (51.6%) 6–10: 32 (34.4%) >10: 13 (14.0%) Dogs: <6: 69 (74.2%) 6–10: 14 (15.1%) >10: 10 (10.8%)
Australia	29 (2.4%)	Rural 3 (10.3%) Urban 7 (24.1%) Suburban 19 (65.5%) Other 0 (0%)	SA 27 (93.1%) Mixed 2 (6.9%) Other 0 (0%)	Private 24 (82.8%) Charity 1 (3.4%) Referral/Uni 4 (13.8%) Other 0 (0%)	<20% insured 29 (100%) >20% insured 0 (0%)	Cats: <6: 13 (44.8%) 6–10: 11 (37.9%) >10: 5 (17.2%) Dogs: <6: 19 (65.5%) 6–10: 3 (10.3%) >10: 7 (24.1%)

DM – Diabetes Mellitus; SA—100% small animal practice; Uni—referral or university practice.

**Table 2:** Description of clinicians’ DM treatment habits.

Parameter	Unit	Value	
		Cats	Dogs
**Proportion of clinicians prescribing twice daily insulin injections**	Number (percentage) of clinicians	980 of 1182 (82.9%)	946 of 1132 (83.6%)
**Starting insulin dose**	U/kg BW/injection: mean ± SD	0.45 ± 0.31	0.60 ± 0.52
**Number of cats/dogs started on new diet at diagnosis**	Median number of cats/dogs (IQR)	9 of 10 (6–10)	7 of 10 (3–9)
**Number of cats/dogs started on oral hypoglycaemic drugs at diagnosis**	Median number of cats/dogs (IQR)	0 of 10 (0–0)	0 of 10 (0–0)

BW—body weight; SD—standard deviation; IQR—interquartile range.

**Table 3:** Answers to questions about main treatment decisions in regards to DM (euthanasia at the time of diagnosis, at 1 month and 1 year).

Question Number	“Typically, in this practice, out of ten cats or dogs newly diagnosed with diabetes mellitus,….” (second part of the question continued below)	Cats: Median (IQR)	Dogs: Median (IQR)
C1/D1	“… how many are euthanased on request of the owner at time of diagnosis?”	1 (0–2)	1 (0–1)
C2/D2	“…how many are euthanased on request of the owner because of not wanting to treat with insulin injections at time of diagnosis?”	1 (0–1)	0 (0–1)
C3/D3	“…and started on insulin injections, in how many is insulin treatment subsequently stopped within 1 month because of lack of success or compliance?”	0 (0–1)	0 (0–1)
C4/D4	“…and started on insulin injections, in how many is insulin treatment subsequently stopped within 1 year because of lack of success or compliance?”	1 (0–2)	1 (0–2)

IQR—interquartile range.

**Table 4:** Answers to questions about quality of life and frequency of complications of DM. Questions C6/D6 as well as question 9 relates to owners’ quality of life and difficulties fitting in twice daily injections, respectively. In question 9, cat and dog owners were not addressed separately.

Question Number	“Typically, in this practice, out of ten cats or dogs newly diagnosed with diabetes mellitus (or their owners),….” (second part of the question continued below)	Cats (Cat Owners): Median (IQR)	Dogs (Dog Owners): Median (IQR)
C5/D5	“…how many achieve a satisfactory quality of life according to the owner once treatment with insulin injections has been started?”	8 (7–9)	8 (7–9)
C6/D6	“…how many of the owners eventually have a satisfactory quality of life and not feel limited in lifestyle because of daily insulin injections?”	8 (6–9)	8 (6–9)
C7/D7	“…how many suffer from an apparent hypoglycaemic crisis (suggestive clinical signs OR recorded blood glucose) at some stage during treatment?“	2 (1–4)	2 (1–3)
C8/D8	“…how many will suffer from an episode of keto-acidosis (DKA) after insulin treatment has been initiated?“	1 (0–2)	1 (0–2)
9	“...how many of the owners report having difficulties fitting in a twice daily injection treatment instead of a once daily injection treatment?” ^a^	2 (1–4)	

IQR—interquartile range.

**Table 5:** Results of multivariate analysis for questions C1, C2, D1 and D2.

Factor		Frequency Euthanasia Newly Diagnosed Diabetic Cats (C1)		Frequency Euthanasia Newly Diagnosed Diabetic Dogs D1		Frequency Euthanasia Newly Diagnosed Diabetic cats—Not Wanting to Inject C2		Frequency Euthanasia Newly Diagnosed Diabetic Dogs—Not Wanting to Inject D2	
		*p*-value	OR (95% CI)	*p*-value	OR (95% CI)	*p*-value	OR (95% CI)	*p*-value	OR (95% CI)
**Country**	UK and Ireland	0.331	0.75 (0.42–1.34)	0.188	0.67 (0.38–1.21)	0.290	0.72 (0.40–1.32)	0.828	0.93 (0.51–1.72)
	USA	0.383	0.83 (0.54–1.27)	0.349	0.81 (0.53–1.25)	0.294	0.79 (0.51–1.23)	0.835	0.95 (0.61–1.50)
	Canada	**0.001**	2.68 (1.53–4.67)	**0.050**	1.75 (1–3.08)	**0.003**	2.36 (1.34–4.16)	**0.014**	2.06 (1.15–3.68)
	Australia	**0.038**	2.26 (1.04–4.88)	0.32	2.38 (1.08–5.27)	0.537	1.28 (0.58–2.83)	0.105	1.97 (0.87–4.46)
	Cont. Europe ^a^	–	^a^	–	^a^	–	^a^	–	^a^
**Location**	Rural	**0.010**	1.6 (1.12–2.28)	**0.001**	1.81 (1.26–2.59)	**0.000**	2.17 (1.51–3.12)	**0.000**	2.17 (1.51–3.13)
	Urban	0.339	1.14 (0.87–1.47)	0.63	1.29 (0.99–1.68)	0.385	1.13 (0.86–1.47)	0.300	1.16 (0.88–1.53)
	Sub-urban ^a^	–	^a^	–	^a^	–	^a^	–	^a^
**Practice type I**	Mixed	**0.009**	1.69 (1.14–2.50)	**0.02**	1.59 (1.07–2.35)	0.987	1.00 (0.67–1.51)	0.312	1.23 (0.82–1.85)
	SA ^a^	–	^a^	–	^a^	–	^a^	–	^a^
**Practice type II**	Referral/Uni	**0.032**	0.6 (0.37–0.96)	0.439	0.83 (0.52–1.33)	**0.015**	0.54 (0.33–0.89)	0.560	0.86 (0.53–1.41)
	Charity	0.424	1.3 (0.68–2.5)	0.101	1.72 (0.90–3.31)	0.129	1.69 (0.86–3.33)	0.597	1.2 (0.61–2.37)
	Private	–	^a^	–	^a^	–	^a^	–	^a^
**% insured**	>20%	0.693	1.11 (0.67–1.82)	0.809	0.94 (0.57–1.56)	0.988	1.00 (0.59–1.68)	0.562	0.86 (0.51–1.45)
	<20% ^a^	–	^a^	–	^a^	–	^a^	–	^a^

CI—confidence interval; ^a^ Reference category; **Bold:** Statistically significant association (*p* ≤ 0.05).

**Table 6:** Results of multivariate analysis for questions C3, C4, D3 and D4.

Factor		Frequency Treatment Stopped Cats <1 Month C3		Frequency Treatment Stopped Dogs <1 Month D3		Frequency Treatment Stopped Cats <1 Year C4		Frequency Treatment Stopped Dogs <1 Year D4	
		*p*-value	OR (95% CI)	*p*-value	OR (95% CI)	*p*-value	OR (95% CI)	*p*-value	OR (95% CI)
**Country**	UK and Ireland	**0.017**	0.47 (0.26–0.88)	**0.026**	0.48 ^b^ (0.25–0.92)	0.307	0.74 (0.41–1.32)	0.136	0.64 (0.36–1.15)
	USA	**0.033**	0.61 (0.28–0.94)	0.148	0.71 (0.44–1.13)	0.614	1.12 (0.73–1.71)	0.490	0.86 (0.56–1.32)
	Canada	**0.017**	0.47 (0.43–0.51)	0.630	0.86 (0.61–1.11)	0.350	1.30 (0.79–1.81)	0.969	1.01 (0.45–1.48)
	Australia	0.318	0.65 (0.68–2.03)	0.623	0.80 (0.33–1.94)	0.939	1.03 (0.48–2.23)	0.767	1.13 (0.51–2.49)
	Cont. Europe ^a^	–	^a^	–	^a^	–	^a^	–	^a^
**Location**	Rural	0.766	1.06 (0.72–1.56)	0.061	1.45 (0.98–2.14)	0.892	0.98 (0.69–1.39)	0.260	1.23 (0.86–1.76)
	Urban	0.786	0.96 (0.72–1.28)	0.322	1.16 (0.86–1.57)	0.451	0.91 (0.70–1.17)	0.440	1.11 (0.85–1.44)
	Sub-urban ^a^	–	^a^	–	^a^	–	^a^	–	^a^
**Practice type I**	Mixed	0.155	1.35 (0.89–2.05)	0.149	1.37 (0.89–2.09)	**0.012**	1.64 (1.12–2.41)	**0.01**	1.67 (1.13–1.47)
	SA ^a^	–	^a^	–	^a^	–	^a^	–	^a^
**Practice type II**	Referral/Uni	0.404	1.23 (0.75–2.01)	0.367	0.78 (0.45–1.34)	**0.045**	1.59 (1.01–2.49)	0.232	1.32 (0.84–2.09)
	Charity	**0.023**	2.18 (1.11–4.27)	**0.002**	2.93 (1.49–5.78)	**0.028**	2.05 (1.08–3.87)	**0.047**	1.92 (1.01–3.66)
	Private ^a^	–	^a^	–	^a^	–	^a^	–	^a^
**% insured**	>20%	0.556	1.18 (0.68–2.03)	0.588	1.17 (0.66–2.08)	0.991	1.00 (0.61–1.65)	0.195	0.72 (0.43–1.19)
	<20% ^a^	–	^a^	–	^a^	–	^a^	–	^a^

CI—confidence interval; ^a^ Reference category; **Bold:** Statistically significant association (*p* < 0.05).

## Supplementary Materials

The following are available online at http://www.mdpi.com/2306-7381/4/2/27/s1, Supplement 1—The Big Pet Diabetes Survey, Supplement 2—Results of univariate analysis for the main treatment decision questions (D/C 1-4).
