# Supplementary material for: The Big Pet Diabetes Survey: Perceived Frequency and Triggers for Euthanasia
Source: Vet Sci. 2017 May 14;4(2):27. doi: 10.3390/vetsci4020027 (PMC5606606; doi:10.3390/vetsci4020027)
Supplement: Supplementary file 1 [file vetsci-04-00027-s001.zip › vetsci-176738-supplement/vetsci-176738-supplementary-final-1.pdf]

Supplementary 1. The Big Pet Diabetes Survey

Introduction

Questionnaire for veterinarians involved in the treatment of dogs and cats with diabetes
Short-term aim:
To gain information on the perceived benefits and shortcomings of insulin treatment in veterinary practices.
Long-term aim:
To use this information to develop superior diabetic treatment and improve the quality of life of diabetic pets and their owners.
Instructions:
Filling in the questionnaire should not take longer than 5-10 minutes of your time. Please note that **only one** VET questionnaire should be submitted per practice/ practice branch. Please pay close attention to words that specify the statement or question (i.e ….. in the **cat**, …… in the **dog**). Please, feel free to provide additional comments at the end of the questionnaire.
Many thanks for your cooperation! With your help we hope to improve the welfare of our diabetic patients.

©The Royal Veterinary College 2007

General Practice Questions

1. What country do you practice in?
- O UK
- O USA
- O Australia
- O Austria
- O Belgium
- O Canada
- O Denmark
- O Belgium
- O Finland
- O France
- O Germany
- O Greece
- O Hungary
- O Italy
- O Ireland
- O Luxembourg
- O Netherlands
- O Norway
- O Poland
- O Portugal
- O Spain
- O Sweden
- O Switzerland
- O Other

If you selected Other, please give details here:

2. My practice is located in a location best described as:
- O Rural
- O Urban
- O Suburban
- O Other

If you selected Other, please give details:

3. My practice is:
- O 100% small animals
- O Mixed
- O Other

If you selected Other, please give details:

©The Royal Veterinary College 2007

4. Typically, in this practice, how many cats and dogs are insured?

	1-10%	11-20%	21-30%	31-40%	41-50%	51-60%	61-70%	71-80%	81-90%	91-100%	Not Applicable
Dogs											
Cats											

5. My practice is a:
- O Private practice
- O Charity
- O Charity and private practice
- O First line university practice
- O Small animal referral practice
- O First line and small animal referral practice
- O Other

If you selected Other, please give details:

Diabetic Treatment Questions (5-10 minutes)

1. Please estimate the number of **cats** newly diagnosed with diabetes mellitus in your practice per year: O 0 O 1 O 2 O 3 O 4 O 5 O 6 O 7 O 8 O 9 O 10 O More than 10

2. Please estimate the number of **dogs** newly diagnosed with diabetes mellitus in your practice per year: O 0 O 1 O 2 O 3 O 4 O 5 O 6 O 7 O 8 O 9 O 10 O More than 10

3. Typically, in this practice, out of 10 **cats** newly diagnosed with diabetes mellitus how many are started on a new diet at time of diagnosis?
O 0 O 1 O 2 O 3 O 4 O 5 O 6 O 7 O 8 O 9 O 10

4. Typically, in this practice, out of 10 **dogs** newly diagnosed with diabetes mellitus how many are started on a new diet at time of diagnosis?
O 0 O 1 O 2 O 3 O 4 O 5 O 6 O 7 O 8 O 9 O 10

5. Typically, in this practice, out of 10 **cats** newly diagnosed with diabetes mellitus how many are started on oral hypoglycaemics (eg glipizide, acarbose) at time of diagnosis? O 0 O 1 O 2 O 3 O 4 O 5 O 6 O 7 O 8 O 9 O 10

©The Royal Veterinary College 2007

6. Typically, in this practice, out of 10 **dogs** newly diagnosed with diabetes mellitus how many are started on oral hypoglycaemics (eg glipizide, acarbose) at time of diagnosis? ○ 0 ○ 1 ○ 2 ○ 3 ○ 4 ○ 5 ○ 6 ○ 7 ○ 8 ○ 9 ○ 10

7. Typically, in this practice, out of 10 **cats** newly diagnosed with diabetes mellitus how many are started on subcutaneous insulin injections at time of diagnosis? ○ 0 ○ 1 ○ 2 ○ 3 ○ 4 ○ 5 ○ 6 ○ 7 ○ 8 ○ 9 ○ 10

8. Typically, in this practice, out of 10 **dogs** newly diagnosed with diabetes mellitus how many are started on subcutaneous insulin injections at time of diagnosis? ○ 0 ○ 1 ○ 2 ○ 3 ○ 4 ○ 5 ○ 6 ○ 7 ○ 8 ○ 9 ○ 10

9. Typically, in this practice, out of 10 **cats** newly diagnosed with diabetes mellitus how many are euthanased on request of the owner at time of diagnosis?
○ 0 ○ 1 ○ 2 ○ 3 ○ 4 ○ 5 ○ 6 ○ 7 ○ 8 ○ 9 ○ 10

10. Typically, in this practice, out of 10 **dogs** newly diagnosed with diabetes mellitus how many are euthanased on request of the owner at time of diagnosis?
○ 0 ○ 1 ○ 2 ○ 3 ○ 4 ○ 5 ○ 6 ○ 7 ○ 8 ○ 9 ○ 10

11. Typically, in this practice, out of 10 **cats** newly diagnosed with diabetes mellitus how many are euthanased on request of the owner **because of not wanting to treat with insulin injections at time of diagnosis**? ○ 0 ○ 1 ○ 2 ○ 3 ○ 4 ○ 5 ○ 6 ○ 7 ○ 8 ○ 9 ○ 10

12. Typically, in this practice, out of 10 **dogs** newly diagnosed with diabetes mellitus how many are euthanased on request of the owner **because of not wanting to treat with insulin injections at time of diagnosis**? ○ 0 ○ 1 ○ 2 ○ 3 ○ 4 ○ 5 ○ 6 ○ 7 ○ 8 ○ 9 ○ 10

©The Royal Veterinary College 2007

13. According to you, how much are **owners** of diabetic animals concerned by the following issues:

	No concern	Little concern	Moderate concern	Great concern	Not Applicable
Hypoglycaemia					
Diabetic ketoacidosis					
Having to inject their animal					
Costs of treatment					
Quality of life of the animal					
Life style changes that the owner has to make					

Any other concerns we might have forgotten?

©The Royal Veterinary College 2007

14. How much do the following issues worry **you as a vet**:

	No concern	Little concern	Moderate concern	Great concern	Not Applicable
Hypoglycaemia					
Diabetic ketoacidosis					
Costs of treatment					
Quality of life of the animal					
Difficulties in obtaining rapid and adequate control					
Difficulties of getting the owner on board with the treatment					

Any other concerns we might have forgotten?

©The Royal Veterinary College 2007

15. If diabetic animals are euthanased/ treatment is stopped/ not started, how important are the following factors:

	Not important	Of little importance	Moderate importance	Great importance	Not Applicable
Costs					
Welfare of pet					
Too much impact on lifestyle of owner					
Injection problems					
Problems obtaining adequate control					
Concurrent disease					
Age of the animal					
Other					

If you identified another factor, please give details:

16. Typically, in this practice, out of 10 **cats** newly diagnosed with diabetes mellitus and started on insulin injections, in how many is insulin treatment subsequently stopped within 1 **month** because of lack of success or compliance?
○ 0 ○ 1 ○ 2 ○ 3 ○ 4 ○ 5 ○ 6 ○ 7 ○ 8 ○ 9 ○ 10

17. Typically, in this practice, out of 10 **cats** newly diagnosed with diabetes mellitus and started on insulin injections, in how many is insulin treatment subsequently stopped within 1 **year** because of lack of success or compliance?
○ 0 ○ 1 ○ 2 ○ 3 ○ 4 ○ 5 ○ 6 ○ 7 ○ 8 ○ 9 ○ 10

18. Typically, in this practice, out of 10 **dogs** newly diagnosed with diabetes mellitus and started on insulin injections, in how many is insulin treatment subsequently stopped within 1 **month** because of lack of success or compliance?
○ 0 ○ 1 ○ 2 ○ 3 ○ 4 ○ 5 ○ 6 ○ 7 ○ 8 ○ 9 ○ 10

©The Royal Veterinary College 2007

19. Typically, in this practice, out of 10 **dogs** newly diagnosed with diabetes mellitus and started on insulin injections, in how many is insulin treatment subsequently stopped within 1 **year** because of lack of success or compliance?
○ 0 ○ 1 ○ 2 ○ 3 ○ 4 ○ 5 ○ 6 ○ 7 ○ 8 ○ 9 ○ 10

20. Typically, in this practice, out of 10 **cats** newly diagnosed with diabetes mellitus, how many achieve a satisfactory quality of life according **to the owner** once treatment with insulin injections has been started? ○ 0 ○ 1 ○ 2 ○ 3 ○ 4 ○ 5 ○ 6 ○ 7 ○ 8 ○ 9 ○ 10 ○ Don't know

21. Typically, in this practice, out of 10 **dogs** newly diagnosed with diabetes mellitus, how many achieve a satisfactory quality of life according **to the owner** once treatment with insulin injections has been started? ○ 0 ○ 1 ○ 2 ○ 3 ○ 4 ○ 5 ○ 6 ○ 7 ○ 8 ○ 9 ○ 10 ○ Don't know

22. Typically, in this practice, out of 10 owners with **cats** newly diagnosed with diabetes mellitus, how many of **the owners** eventually have a satisfactory quality of life and not feel limited in lifestyle because of daily insulin injections?
○ 0 ○ 1 ○ 2 ○ 3 ○ 4 ○ 5 ○ 6 ○ 7 ○ 8 ○ 9 ○ 10 ○ Don't know

23. Typically, in this practice, out of 10 owners with **dogs** newly diagnosed with diabetes mellitus, how many of **the owners** eventually have a satisfactory quality of life and not feel limited in lifestyle because of daily insulin injections?
○ 0 ○ 1 ○ 2 ○ 3 ○ 4 ○ 5 ○ 6 ○ 7 ○ 8 ○ 9 ○ 10 ○ Don't know

24. Typically, in this practice, out of 10 **cats** newly diagnosed with diabetes mellitus, how many suffer from an apparent hypoglycaemic crisis (suggestive clinical signs OR recorded blood glucose) at some stage during treatment?
○ 0 ○ 1 ○ 2 ○ 3 ○ 4 ○ 5 ○ 6 ○ 7 ○ 8 ○ 9 ○ 10

25. Typically, in this practice, out of 10 **dogs** newly diagnosed with diabetes mellitus, how many suffer from an apparent hypoglycaemic crisis (suggestive clinical signs OR recorded blood glucose) at some stage during treatment?
○ 0 ○ 1 ○ 2 ○ 3 ○ 4 ○ 5 ○ 6 ○ 7 ○ 8 ○ 9 ○ 10

26. In your experience, out of 10 diabetic **cats** seen in your practice how many will suffer from an episode of keto-acidosis (DKA) **after** insulin treatment has been initiated? ○ 0 ○ 1 ○ 2 ○ 3 ○ 4 ○ 5 ○ 6 ○ 7 ○ 8 ○ 9 ○ 10

©The Royal Veterinary College 2007

27. In your experience, out of 10 diabetic **dogs** seen in your practice how many will suffer from an episode of keto-acidosis (DKA) **after** insulin treatment has been initiated? O 0 O 1 O 2 O 3 O 4 O 5 O 6 O 7 O 8 O 9 O 10

28. Are owners more likely to opt for insulin injections under the following circumstances?

	Definitely not	Maybe	Probably	Definitely yes	Don't know
When the animal is insured					
If they themselves or close family or friends are diabetic					

29. Do you tend to start once daily (SID) or twice daily (BID) insulin injections when starting insulin treatment in a cat?

O SID
O BID

30. Do you tend to start once daily (SID) or twice daily (BID) insulin injections when starting insulin treatment in a dog?

O SID
O BID

31. Typically, in this practice, out of 10 owners with animals newly diagnosed with diabetes mellitus, how many of **the owners** report having difficulties fitting in a twice daily injection treatment instead of a once daily injection treatment?

O 0 O 1 O 2 O 3 O 4 O 5 O 6 O 7 O 8 O 9 O 10

©The Royal Veterinary College 2007

32. According to you how important are the following compliance issues encountered in your practice?

	Not important	Of little importance	Moderate importance	Great importance
**Owner** having injection difficulties				
**Cat** causing injection difficulties				
**Dog** causing injection difficulties				
Inappropriate insulin storage				
Injections not given at right times				
Owner does not stick to feeding protocol				
Other				

If you identified another factor, please give details:

33. Would you treat your own **cat** with daily insulin injections if diagnosed with diabetes?

- O Definitely not 0
- O Maybe 1
- O Probably 2
- O Definitely yes 3
- O Don't know 4

34. Would you treat your own **dog** with daily insulin injections if diagnosed with diabetes?

- O Definitely not
- O Maybe
- O Probably
- O Definitely yes
- O Don't know

©The Royal Veterinary College 2007

35. What is your approximate starting insulin dose per kilogram in **cats**?

36. What is your approximate starting insulin dose per kilogram in **dogs**?

37. Any comments you would like to make on any of the discussed topics?

©The Royal Veterinary College 2007
