# Supplementary material for: The Big Pet Diabetes Survey: Perceived Frequency and Triggers for Euthanasia
Source: Vet Sci. 2017 May 14;4(2):27. doi: 10.3390/vetsci4020027 (PMC5606606; doi:10.3390/vetsci4020027)
Supplement: Supplementary file 1 [file vetsci-04-00027-s001.zip › vetsci-176738-supplement/vetsci-176738-supplementary-final-2.pdf]

Supplement 2: Results of univariate analysis for the main treatment decision questions (D/C 1-4)

Table S1. Univariate analysis of euthanasia related questions. Grey shading indicates statistical significance.
D1/C1: "Typically, in this practice, out of 10 dogs/cats newly diagnosed with diabetes mellitus, how many are
euthanased on request of the owner at time of diagnosis?"
D2/C2: ""Typically, in this practice, out of 10 dogs/cats newly diagnosed with diabetes mellitus, how many are
euthanased on request of the owner because of not wanting to treat with insulin injections at time of diagnosis?".

Variable	Group (n=number of respondents)	Median [IQR]				P-value [a]			
		D 1	D 2	C 1	C 2	D 1	D 2	C 1	C 2
Country	UK & Ireland (n=168)	1 [0-1]	0 [0-1]	1 [0-1]	1 [0-1]	<0.001	0.001	<0.001	<0.001
	USA (n=790)	1 [0-1]	0 [0-1]	1 [0-1]	1 [0-1]				
	Canada (n=92)	1 [1-2]	1 [0-1.75]	1 [1-2]	1 [0-2]				
	Australia (n=27)	1 [1-2]	1 [0-2]	1 [0.5-2]	1 [0-2]				
	Cont. Europe (n=90)	1 [0-2]	0 [0-1]	1 [0-2]	1 [0-1]				
Practice location	Rural (n=156)	1 [0-2]	1 [0-2]	1 [0-2]	1 [0-2]	<0.001	<0.001	<0.001	<0.001
	Urban (n=363)	1 [0-1]	0 [0-1]	1 [0-2]	1 [0-1]				
	Suburban (n=637)	1 [0-1]	0 [0-1]	1 [0-1]	0 [0-1]				
Practice type I	Mixed (n=114)	1 [0-2]	1 [0-1]	1 [0.25-2]	1 [0-2]	<0.001	0.002	<0.001	0.041
	SA (n=1022)	1 [0-1]	0 [0-1]	1 [0-1]	1 [0-1]				
Practice type II	Referral/Uni (n=79)	1 [0-1]	0 [0-1]	1 [0-1]	0 [0-1]	0.214	0.412	0.179	0.008
	Charity (n=35)	1 [0-2]	1 [0-1]	1 [0-2]	1 [0-1.5]				
	Private (n=1015)	1 [0-1]	0 [0-1]	1 [0-2]	1 [0-1]				
% insured	>20% insured (n=130)	1 [0-1]	0 [0-1]	1 [0-2]	0.5 [0-1]	0.121	0.167	0.716	0.438
	<20% insured (n=1042)	1 [0-1]	0 [0-1]	1 [0-2]	1 [0-1]				

IQR – interquartile range; Cont. Europe – Continental Europe; SA – 100% small animal practice; Uni – university hospital; [a] Kruskall-Wallis Test

Table 2. Percentages of 0/10 and 2/10 responses to euthanasia related questions D1/C1 and D2/C2.

Variable	Group (n=number respondents)	% 0 out of 10 / % 2 out of 10			
		D 1	D 2	C 1	C 2
Country	UK & Ireland (n=168)	46.4 / 9.5	57.1 / 8.9	39.0 / 20.1	47.9 / 10.3
	USA (n=790)	46.3 / 11.4	56.0 / 6.7	38.7 / 13.4	48.9 / 8.0
	Canada (n=92)	23.9 / 15.2	37.0 / 12.0	15.1 / 28.0	30.1 / 19.4
	Australia (n=27)	29.6 / 18.5	48.1 / 14.8	24.1 / 31.0	44.8 / 20.7
	Cont. Europe (n=90)	44.4 / 17.8	56.7 / 7.8	40.9 / 14.0	47.8 / 10.9
Practice location	Rural (n=156)	32.5 / 14.3	37.4 / 13.5	25.2 / 20.0	32.7 / 15.4
	Urban (n=363)	41.9 / 12.8	54.6 / 7.8	36.9 / 17.9	47.7 / 10.7
	Suburban (n=637)	48.6 / 11.0	58.8 / 6.2	39.7 / 13.8	50.2 / 8.0
Practice type I	Mixed (n=114)	30.8 / 12.8	42.2 / 12.1	25.0 / 21.6	39.5 / 14.0
	SA (n=1022)	44.8 / 12.2	55.3 / 7.4	37.0 / 15.4	47.2 / 9.5
	Referral/Uni (n=79)	49.4 / 12.7	62.0 / 10.1	46.8 / 11.4	63.8 / 7.5

Practice type II	Charity (n=35)	31.4 / 11.4	48.6 / 5.7	31.4 / 17.1	33.3 / 15.2
	Private (n=1015)	44.0 / 12.2	54.0 / 7.7	35.8 / 16.1	46.0 / 9.7
% insured	>20% insured (n=130)	48.5 / 9.0	61.2 / 9.7	38.6 / 16.7	50.0 / 8.5
	<20% insured (n=1042)	43.4 / 12.4	53.6 / 7.4	36.4 / 15.8	46.6 / 9.9

Cont. Europe – Continental Europe; SA – 100% small animal practice; Uni – university hospital

Table 3. Univariate analysis of euthanasia related questions. Grey shading indicates statistical significance.
D3/C3: "Typically, in this practice, out of 10 dogs/cats newly diagnosed with diabetes and started on insulin injections, in how many is insulin treatment subsequently stopped within 1 month because of lack of success or compliance? "
D4/C4: "Typically, in this practice, out of 10 dogs/cats newly diagnosed with diabetes and started on insulin injections, in how many is insulin treatment subsequently stopped within 1 year because of lack of success or compliance?"

**Variable**	**Group (n=number respondents)**	**Median [IQ range]**				**P-value**			
		**D 3**	**D 4**	**C 3**	**C 4**	**D3**	**D 4**	**C 3**	**C 4**
Country	UK & Ireland (n=168)	0 [0-1]	0.5 [0-1]	0 [0-1]	1 [0-2]	0.249	0.055	0.084	0.393
	USA (n=790)	0 [0-1]	1 [0-2]	1 [0-1]	1 [0-1]				
	Canada (n=92)	0 [0-1]	1 [0-2]	0 [0-1]	1 [0-2]				
	Australia (n=27)	0 [0-1]	1 [0-2]	0 [0-1]	1 [0-2]				
	Cont. Europe (n=90)	0 [0-1]	1 [0-2]	0 [0-2]	1 [0-2]				
Practice location	Rural (n=156)	0 [0-1]	1 [0-2]	0 [0-1]	1 [0-2]	0.032	0.052	0.608	0.381
	Urban (n=363)	0 [0-1]	1 [0-1]	0 [0-1]	1 [0-2]				
	Suburban (n=637)	0 [0-1]	1 [0-2]	0 [0-1]	1 [0-2]				
Practice type I	Mixed (n=114)	0 [0-1]	1 [0-2]	0 [0-1]	1 [0-2]	0.015	0.001	0.046	0.014
	SA (n=1022)	0 [0-1]	1 [0-2]	0 [0-1]	1 [0-2]				
Practice type II	Referral/Uni (n=79)	0 [0-1]	1 [0-2]	0 [0-1]	1 [0-2]	0.010	0.172	0.082	0.156
	Charity (n=35)	1 [0-1]	1 [0-2]	1 [0-1]	1 [1-2]				
	Private (n=1015)	0 [0-1]	1 [0-2]	0 [0-1]	1 [0-2]				
% insured	>20% insured (n=130)	0 [0-1]	0 [0-1]	0 [0-1]	1 [0-2]	0.335	0.002	0.980	0.119
	<20% insured (n=1042)	0 [0-1]	1 [0-2]	0 [0-1]	1 [0-2]				

IQR – interquartile range; Cont. Europe – Continental Europe; SA – 100% small animal practice; Uni – university hospital; [a] Kruskall-Wallis Test
